# Digital Health Intervention to Promote Lifelong Specialized Care in Adults With Congenital Heart Disease: Theory-Driven Community Co-Designed Study

**DOI:** 10.2196/75867

**Published:** 2026-06-23

**Authors:** Anushree Agarwal, Joseph Valente, Karina Buenrostro, Katelyn Macholl, Juhi Mehta, Keerthana Reddy, Karina Manayan, Parang Kim, Aleah Sparks, Kunyi Li, Pranav Ahuja, Kevin Sun, Kimberly Payton, Mark D Norris, Katia Bravo-Jaimes, Leigh Reardon, Philip Moons, Megumi J Okumura, Gregory M Marcus, Michelle Gurvitz

**Affiliations:** 1 Adult Congenital Heart Disease Section, Division of Cardiology, Associate Professor of Medicine, University of California, San Francisco, 500 Parnassus Avenue, M-1177B, Box 0124, San Francisco, 94143-0124, United States, 1 415-353-3817, 1 415-353-2528; 2 Team Uncle Joe, Katy, TX, United States; 3 Department of Medicine, Division of Cardiology, University of California, San Francisco, San Francisco, CA, United States; 4 Division of Pediatric Cardiology, University of Miami, Miami, FL, United States; 5 Golden Gate Regional CenterSan Francisco, CA, United States; 6 Senior Patient Advocate and 1st Vice East County NAACP, Parent of an adult with congenital heart disease, East County NAACP, Pittsburg, CA, United States; 7 Department of Pediatrics, Division of Cardiology, University of Michigan, Ann Arbor, United States; 8 Department of Cardiovascular Medicine, Mayo Clinic in Florida, Jacksonville, United States; 9 Division of Pediatric Cardiology, University of California, Los Angeles, Los Angeles, United States; 10 KU Leuven Department of Public Health and Primary Care, KU Leuven, Leuven, Belgium; 11 Sahlgrenska Academy, University of Gothenburg Centre for Person-Centered Care (GPCC), University of Gothenburg, Gothenburg, Sweden; 12 Department of Pediatrics and Child Health, University of Cape Town, Cape Town, South Africa; 13 General Internal Medicine and PRL Institute for Health Policy Studies, Departments of Pediatrics and Internal Medicine, Division of General Pediatrics, University of California, San Francisco, San Francisco, United States; 14 Department of Cardiology, Boston Children's Hospital, Brigham and Women's Hospital, Harvard Medical School, Boston, MA, United States

**Keywords:** congenital heart defects, adult, digital health, mobile apps, health behavior, patient-centered care, community-based participatory research, implementation science

## Abstract

**Background:**

Adults with congenital heart disease frequently experience gaps in lifelong adult congenital heart disease (ACHD) specialty care, leading to preventable complications, hospitalizations, and premature mortality. However, effective, scalable, accessible, and sustainable strategies to reduce these gaps are lacking. Digital health offers potential solutions but requires a rigorous scientific approach in its design to address the needs of the target population.

**Objective:**

This study aims to describe the development of a theory-based, community co-designed digital health intervention to improve lifelong ACHD care.

**Methods:**

We integrated theory-based behavioral frameworks, semistructured qualitative interviews with patients and clinicians, and a community-engaged approach to develop a digital health intervention for ACHD. The primary behavioral target was completing an ACHD specialist appointment. We conducted Capability, Opportunity, Motivation, and Behavior (COM-B)–guided semistructured interviews with patients with ACHD and clinicians to identify barriers to specialized care amenable to a digital intervention, and patient-centered goals for the digital tool. The community partners helped develop key intervention objectives, create a theory-driven framework, and specify how each intervention component targets specific COM-B barriers.

**Results:**

We interviewed 54 participants (n=37 patients with ACHD, and n=17 clinicians) and engaged 21 community partners representing 4 advocacy organizations. Design objectives emphasized addressing patient loneliness, ensuring accessibility and credibility, and enabling scalability while centering patient perspectives. Participants identified 4 priorities: providing credible resources, uplifting patient voices, customizing to patient needs, and centering positivity and joy. The digital tool, named by community partners as Empower My Congenital Heart (EMCH), was designed within the web- and mobile-based, Apple- and Android-compatible, Eureka Digital Research platform (University of California, San Francisco). Key intervention components included educational modules, peer support, appointment planning nudges, and a digital medical passport. The EMCH’s theory-driven framework specifies how each intervention component targets specific COM-B barriers to specialized ACHD care.

**Conclusions:**

The theory-driven, community co-designed EMCH digital tool provides a scalable approach to promote lifelong ACHD specialist care. Ongoing process evaluation and a planned randomized controlled trial will assess acceptability, engagement, and effectiveness in reducing care gaps. If proven effective, EMCH has the potential to prevent complications, emergency hospitalizations, and mortality affecting the majority of adults with congenital heart disease.

## Introduction

Adult congenital heart disease (ACHD) is rapidly growing in the adult population [1,2]. The American Heart Association and American College of Cardiology guidelines provide recommendations on the frequency and intervals at which patients with ACHD should receive lifelong care from clinicians specialized in managing ACHD [3,4]. Despite this, up to 85% of patients with ACHD experience gaps in receiving specialized care throughout their adult lives [5-8]. Those with care gaps are at a higher risk of poor outcomes, including emergent admissions, need for urgent cardiac procedures, and mortality [9-11]. Thus, there is a need to identify accessible, scalable, and sustainable strategies to reduce care gaps and improve outcomes for patients with ACHD.

Individual-, provider-, and system-level barriers contribute to gaps in lifelong ACHD care [5,12,13]. Interventions, such as education and support for navigating the health system, can reduce these gaps [14-16]. However, existing interventions are clinic-based (nurse-led education, transition clinics); limited to single-center studies of 16‐ to 21-year-olds focused on transfer from pediatric to ACHD care; and are resource-intensive, inconsistently implemented, and constrained by reimbursement, clinic time, and missed visits [17-19].

Web- and mobile-based solutions offer a scalable way to overcome these barriers by engaging patients outside of clinic visits [20,21]. Although 94% of patients with ACHD use smartphones and most report openness to app-based solutions [22], existing congenital heart disease (CHD) digital interventions focus on care transition readiness in adolescents and young adults [23] or general patient monitoring and education [24]. None systematically address the establishment and maintenance of lifelong ACHD care across the full adult lifespan using theory-driven behavior change frameworks. Effective and scalable strategies to support all adults (≥18 y), including those who did not transfer from pediatrics or were later lost from care, in establishing and maintaining ACHD care remain unknown.

App-based interventions grounded in evidence-based theoretical frameworks [21,25] are more robust, adaptable, and effective in achieving lasting behavior change in real-world settings, not just in ideal research environments [26,27]. Designing interventions to change behaviors also requires an understanding of the perspectives and psychosocial contexts of the people who will use them—a concept fundamental to community-engaged research [28-30]. Combining theory- and community-driven approaches is vital to ensure that interventions are usable, acceptable, and move beyond *what* works to *how* to make it work in diverse, complex environments [27,31].

Thus, the aim of this paper is to describe a theory- and community-driven approach to designing a scalable digital health intervention to promote ACHD care. The methods and findings can inform others developing interventions to improve outcomes for ACHD and other chronic, lifelong conditions.

## Methods

### Intervention Development Process

#### Overview

**Figure 1.**
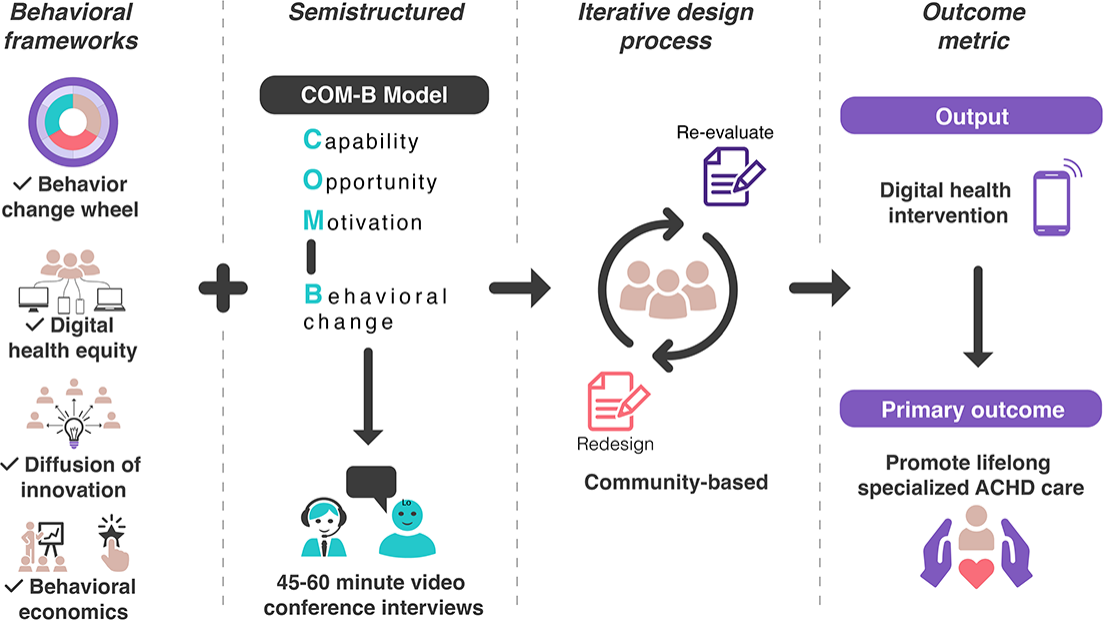
Overview of the digital health intervention planning and development process. Behavioral frameworks (Behavior Change Wheel, Digital Health Equity, Diffusion of Innovation, and Behavioral Economics) were integrated with semistructured interviews and an iterative design process with the community partners. This process optimized the development of a digital health intervention with key objective to empower patients in navigating their congenital heart disease and promote lifelong specialized care. ACHD: adult congenital heart disease.

We began by establishing guiding principles for intervention design (Textbox 1). We then integrated behavioral frameworks with semistructured interviews and community-engaged research to identify and refine intervention components for promoting ACHD care (Figure 1).

Textbox 1. **Guiding philosophy for the design of an adult congenital heart disease (ACHD) digital health intervention.**

**Characteristics designed to drive evidence-based approach**

- Explicitly evidence-based, using scientific rationale for behavioral theories
- Expert guidance for content creation (eg, adult congenital heart disease follow-up guidelines)
- Person- and theory-informed approach at all stages of design and evaluation to meet individual needs of the target adult congenital heart disease population
- Noncommercial, developed by the named team of medical and behavior change experts

**Characteristics designed to encourage uptake and long-term engagement**

- Nimble, user-friendly interface with layperson-oriented messaging, leveraging existing tested capabilities and proven methods of the digital research platform
- Simplicity, with direct, concise, and actionable messaging designed to foster participants’ capabilities, opportunities, and motivation
- Proactive, repetitive content delivery using nudge principles rather than relying on patients to seek out information, enhancing its reach and impact
- Mechanisms for modeling and sharing experiences within the user community, aimed at boosting self-efficacy and promoting positive affect among participants

#### Behavioral Frameworks

The study team chose 4 evidence-based behavioral frameworks that complemented each other. First, the Behavior Change Wheel (BCW) theoretical model was used to specify the target behavior, identify the barriers and enablers of the behavior, and select the means by which the intervention can change behaviors [32,33]. To make the intervention equitable and relevant to diverse patients with ACHD, we incorporated the principles of Digital Health Equity, Diffusion of Innovation, and Behavioral Economics (Table 1) [34-36].

The BCW framework is based on multiple models of health behavior and has been shown to be effective in various cardiac and noncardiac chronic conditions [37-40] but has not yet been used in ACHD. The core driver in BCW (or hub of the wheel) is the Capability, Opportunity, Motivation, and Behavior (COM-B) model, which consists of three necessary conditions for a given “Behavior” to occur: (1) “Capability” (psychological or physical), (2) “Opportunity” (physical or social), and (3) “Motivation” (reflective or automatic). The BCW framework then supports the selection of intervention functions and policy categories. Intervention function refers to broad categories of ways an intervention can change a behavior. The 9 intervention functions described in BCW include education, persuasion, incentivization, coercion, training, restrictions, environmental restructuring, modeling, and enablement. Policy categories comprise the final outer layer, or the wheel’s rim, and help identify the types of policy categories one may wish to consider to further influence the drivers of behaviors (COM-B).

**Table 1.** Theoretical frameworks for designing ACHD^a^ digital intervention.

Frameworks	Summary of the theoretical frameworks
Behavioral Change Wheel [32]	Define health problem in behavioral terms (eg, scheduling and completing ACHD specialist visit) Understand determinants of behavior (Capability, Opportunity, Motivation for Behavior Change model) Link determinants of behavior to identify domains most likely to influence behavior Select intervention functions that are means by which an intervention changes behavior (ie, education, enablement, persuasion, or training)
Digital Health Equity [34]	Apply the following concepts: Health literacy: include relevant content: assume no background knowledge of participants, avoid cognitive burden from too much information, and use simple numbers and percentages to develop content Readability: use plain and clear language tested with target population Ease of use: accessible on various platforms, format conducive to comprehension; content appeals to users of different identities and backgrounds
Diffusion of Innovation [35]	Engaging all levels of adopters (highly engaged and less engaged patients with CHD^b^) and CHD champions in the design, adoption, and dissemination of the intervention
Behavioral Economics [36]	Nudges and default settings of the digital tool alter behavior and facilitate decision-making process

a ACHD: adult congenital heart disease.

b CHD: congenital heart disease.

#### Semistructured Interviews

The semistructured interviews for this study were part of a larger qualitative study that was conducted to identify barriers and enablers for lifelong specialized ACHD care, described in detail separately [12]. For this study, we used COM-B as a guide to identify barriers amenable to a digital intervention and the patient-centered goals for the digital tool. Briefly, we recruited patients with ACHD (≥18 y) who spoke English or Spanish and could provide informed consent. Initially, patients were recruited from University of California, San Francisco (UCSF) using chart review and purposefully sampled [41] to recruit those with more than 3-year gaps in ACHD specialist care during their adulthood. Once we achieved thematic saturation [42] at UCSF, we recruited non-UCSF patients with convenience and snowball sampling [41] to have representation from all regions of the United States (Northeast, Southeast, Midwest, Southwest, and West). All patients underwent an informed consent process and completed a demographic questionnaire at the end of their interview. Clinicians included adult congenital cardiology and pediatric cardiology nurses, doctors, coordinators, board-certified patient advocates, and program leaders. Clinicians were recruited from UCSF and outside UCSF using a similar approach of snowballing and thematic saturation to have representation from all regions. Separate interview guides were developed for patients and clinicians using content domains within the COM-B framework and to explore opinions toward a digital tool and its key features (Tables S1 and S2 in Multimedia Appendix 1). Interviews were conducted over Zoom, each lasting 45 to 60 minutes, audio-recorded, and professionally transcribed.

#### Community-Engaged Approach

The goal of the research team in recruiting community advisory board (CAB) partners was to represent diverse perspectives in ACHD care. We used convenience sampling to recruit patients from UCSF and snowball sampling to recruit patients outside UCSF, as well as clinicians and advocacy representatives. CAB partners were involved in all aspects of intervention planning, design, and implementation, including recruitment, reviewing interview findings, finalizing tool features and branding, sharing personal experiences, and iterative prototype testing. One patient partner (JV) led the creation of a design guide (Table S3 in Multimedia Appendix 1) to ensure consistency and uniformity in materials, built the study website, and developed strategies to elevate participant experience. CAB members also contributed as coauthors in scientific and community writing and dissemination. CAB members provided ongoing input through monthly hour-long meetings. Meeting discussions were documented in study logs, iteratively integrated with interview data, and analyzed by the research team to identify key themes and design recommendations.

### Data Analysis

We conducted thematic analysis of all semistructured interviews using an inductive approach that was subsequently mapped to the COM-B framework. Codes emerged organically from participant narratives and were then organized according to COM-B domains (capability, opportunity, motivation). The coding team consisted of AA (ACHD cardiologist with qualitative research training), MJO (pediatric-to-adult transition specialist and qualitative researcher), 1 medical student (K Macholl), and 3 research assistants (KB, JM, PA). All team members completed structured training in the COM-B or TDF framework application and qualitative coding principles, including group practice coding of pilot transcripts to establish shared code definitions. The multicoder consensus process, overseen by AA and MJO, ensured coding rigor while providing methodological training for less experienced team members. Two team members independently coded each transcript in Microsoft Word to identify themes and subthemes related to barriers and facilitators to ACHD care and toward key aspects of the digital tool. After independent coding, coders (AA, K Macholl, KB, JM, PA) met in iterative group sessions to compare coded transcripts and discuss discrepancies. We prioritized resolving discrepancies through consensus discussion among the coding team, rather than quantitative intercoder reliability metrics. The finalized codes were compiled into a single coded transcript for each interview. We then created a structured data matrix using rapid qualitative analysis [43], an action-oriented method for qualitative data analysis conducted in Microsoft Excel, to efficiently produce results for real-world interventions. Coded data from finalized transcripts were transferred into the matrix, which organized codes across participants, allowing systematic cross-case comparison. Sample codebook entries showing how themes were mapped to COM-B and an excerpt of the rapid qualitative analysis matrix structure are provided in Tables S4 and S5 respectively in Multimedia Appendix 1. We summarized the matrix findings via iterative group meetings (AA, K Macholl, KB, JM, PA, MJO). Through ongoing, iterative reviews of CAB meeting discussions documented in study logs, we informed, planned, and refined the intervention components concurrent with their development.

### Ethical Considerations

Ethical approval for this study was obtained from the UCSF institutional review board (IRB number 22‐36667). Verbal informed consent was obtained from interview participants. The National Clinical Trial number for this study is NCT06581484. All interview participants and CAB members were offered US $50 per hour in the form of an electronic gift card to compensate them for their time.

## Results

### Study Population

We interviewed 54 participants (n=37 patients and n=17 clinicians) (Table 2) and partnered with 21 CAB members. The CAB comprised 11 patients or family members who are racially diverse and from all regions of the United States, 6 pediatric or ACHD physicians, 1 social worker, 1 ACHD nurse, 2 nurse practitioners, and 6 advocacy representatives, some of whom represent more than 1 role. Advocacy organizations included the Adult Congenital Heart Association, Conquering CHD, Mended Hearts, and Team Uncle Joe [44-47].

**Table 2.** Characteristics of study participants.

Characteristic	Patients (n=37)	Clinicians (n=17)
Age (y), median (IQR)	32 (18‐65)	—^a^
Female, n (%)	21 (57)	—
Race or ethnicity, n (%)		
Asian	8 (21)	—
Black or African American	6 (15)	—
Hispanic or Latino	7 (18)	—
White	13 (35)	—
Other or multiple	3 (8)	—
Education: less than or equal to some college, n (%)	14 (41)	—
Care gap ≥3 years, n (%)	13 (35)	—
Recruitment source, n (%)		
UCSF^b^	27 (73)	9 (52)
Community or snowball	10 (27)	8 (48)
Clinician roles, n (%)		
ACHD^c^ or pediatric cardiologists	—	8 (47)
Nurses or NPs^d^	—	6 (35)
Other (coordinators, advocates)	—	3 (18)

a Not applicable.

b UCSF: University of California San Francisco.

c ACHD: adult congenital heart disease.

d NP: nurse practitioner.

### CHD-Related Unique Challenges to Inform the Digital Intervention Design and Features

The challenges uniquely faced by patients with ACHD centered around users’ perspectives, loneliness, feasibility, accessibility, credibility, and scalability (Table 3). They then helped identify design objectives and intervention features that could address these challenges. The interview participants also identified four patient-centered goals to consider during the design of the digital tool (Table S6 in Multimedia Appendix 1): (1) easy access to credible resources (How can we make it easier to connect patients with pre-existing resources?); (2) uplifting of patient voices (How can we uplift patient voices to create a personal connection?); (3) customization to patient needs (How can we cater to different needs within the community?); and (4) centering positivity and joy (How can we bring positivity and joy into our tool?).

**Table 3.** Digital tool design objectives and features for adult congenital heart disease (ACHD) specialized care.

Key challenge	Design objective	Key features	Evidence base
Perspectives: Patients may not understand the need for specialist care when feeling well; prefer to avoid thinking about their heart	Promote well-being rather than illness management	Empowering tone positioning patients as active participants; simple, concise interventions (eg, clarifying defect vs disease); user-driven engagement with personally relevant content; building motivation from first contact	Interviews + CAB^a^
Loneliness: Patients feel isolated; unsure how their condition compares to others	Build community	Peer empowerment quotes providing practical guidance; user story sharing; links to community events and peer connections	Interviews + CAB
Feasibility: Risk for the intervention to be overly complex given diverse patient needs and resource constraints	Efficient, multiphase design	Phased development targeting key care gap drivers; features addressing multiple outcomes simultaneously; balance between broad applicability and personalized relevance; continuous adaptation based on user feedback	CAB
Accessibility: Young adults with competing priorities need quick, convenient access	Enable easy, timely, nonintrusive access	Brief, actionable content (few minutes to complete); mobile-optimized display; automated nudge delivery via email, texting, or push notifications; small information bursts over time	Interviews + CAB
Credibility: Abundance of information creates overwhelm; unclear what sources are reliable	Use credible, curated sources	Partnership with advocacy organizations for content curation; easy-to-understand formatting; iterative tailoring based on user feedback	Interviews + CAB
Scalability: Variability in age, health literacy, and prior engagement creates diverse needs	Design for diverse needs	Cross-platform web or mobile access (Android or iOS); low-bandwidth content; materials addressing both activated and nonactivated patients; anonymous story sharing; community building for current and future generations	CAB, guided by interviews

a CAB: community advisory board.

### Theory-Driven Intervention Components

**Figure 2.**
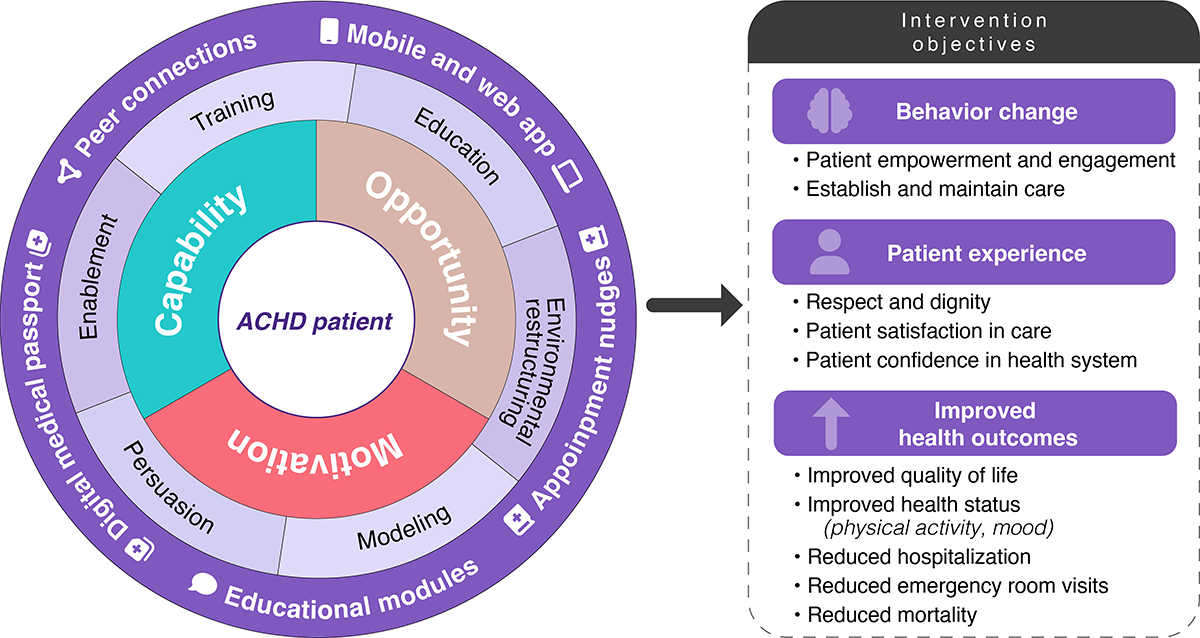
Theoretical framework hypothesizing the impact of digital health intervention components on outcomes. The intervention components (educational modules, digital medical passport, peer connections, and appointment nudges), delivered via web and mobile app, function as tools for education, training, enablement, persuasion, modeling, and environmental restructuring to enhance the Capability, Opportunity, and Motivation (COM-B) domains of patients with ACHD. We hypothesize that these components will support patient activation and engagement skills and lifelong specialized ACHD care, ultimately improving patient experience and health outcomes. ACHD: adult congenital heart disease.

Using the BCW framework, we mapped barriers to specialized care (identified through interviews and CAB discussions) to behavioral targets and intervention functions (Table 4). This systematic approach enabled us to select 6 BCW functions (education, training, environmental restructuring, modeling, persuasion, and enablement) and translate them into specific intervention components. For example, educational modules with analogies explaining CHD conditions address capability barriers (lack of knowledge), while peer narratives simultaneously address social opportunity barriers (modeling self-advocacy) and motivation barriers (building confidence and hope). We further mapped these intervention components to intervention objectives to develop a theoretical framework showing how intervention functions are hypothesized to impact outcomes (Figure 2).

**Table 4.** Capability, Opportunity, Motivation for Behavior Change (COM-B) and Behavior Change Wheel (BCW) framework for the digital intervention to promote specialized ACHD^a^ care.

COM-B and component	Identified barriers (from interviews and CAB^b^)	Behavioral targets (what needs to change?)	BCW intervention functions^c^	Intervention components within the digital tool
Capability				
Psychological	Lack of knowledge about CHD^d^; unable to find ACHD specialists; forget to schedule routine visits	Increase knowledge, help find ACHD specialists, reassurance	Education, Enablement, Persuasion	Educational modules; ACHD Clinic Directory link; ACHD appointment nudges
Opportunity				
Physical	Difficulty keeping all medical history centralized and easily available	Provide secure, easily accessible location	Environmental restructuring, Enablement	Digital medical passport with medical history and provider information
Social	Unable to advocate or connect with physicians; feeling “lonely” given CHD is rare	Prepared to advocate	Modeling, Enablement	Peer narratives demonstrating self-advocacy; peer connection opportunities through community event listings
Motivation				
Reflective	Belief in unmet needs (eg, family planning, travel, drugs, work/school); social stigma and low confidence related to scars	Reassurance that needs can be addressed with the ACHD team	Education, Training or Modeling	Educational content on life topics (pregnancy, career); peer success stories
Automotive	Fear of being dismissed	Foster optimism and address readiness	Persuasion, Modeling	Positive framing; peer narratives building confidence

a ACHD: adult congenital heart disease.

b CAB: community advisory board.

c “Functions” in the COM-B model are as follows: Education, Persuasion (induce positive feelings or stimulate action), Training (impart skills), Environmental restructuring (change the physical or social context), Modeling (examples to aspire to or imitate), and Enablement (reduce barriers).

d CHD: congenital heart disease.

### Design of the Digital Tool

#### Digital Research Platform

The digital tool was developed on the Eureka Research Platform (University of California, San Francisco), a Health Insurance Portability and Accountability Act–secure, National Institutes of Health (NIH)−supported infrastructure enabling integrated recruitment, consent, data collection, and intervention delivery [48]. Participants enroll remotely using QR codes or web links and consent electronically (UCSF IRB number 22‐36667; Table S7 in Multimedia Appendix 1).

#### Branding

The community partners discussed various options for the name and logo of the digital tool and narrowed them down to 4 potential ones: My Congenital Heart Care, Empower My Congenital Heart, My Congenital Heart Guide, and Uplift My Congenital Heart. The CAB members suggested including an arrow in the logo to reflect the primary goal of uplifting and empowering patients. Red, blue, and purple colors were chosen to reflect the diversity within CHD lesions and their uniqueness from other heart diseases. The design team developed 4 branding prototypes (Figure S1 in Multimedia Appendix 1). Through CAB voting, “Empower My Congenital Heart (EMCH)” with an integrated heart-and-arrow logo (option B-2) was selected.

#### Participant Flow Within EMCH

**Figure 3.**
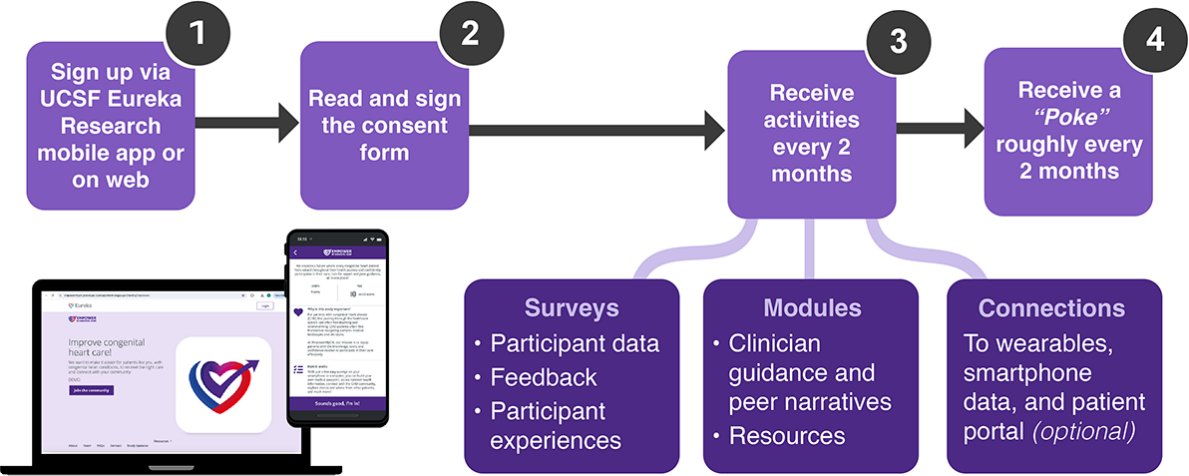
Participant flow through Empower My Congenital Heart (EMCH). EMCH is accessible via the UCSF Eureka Research mobile app or web platform. Participants first create a UCSF Eureka Research account (step 1) and then review and electronically sign the EMCH consent form (step 2). Following consent, participants receive study activities every 2 months (step 3), including surveys (participant data, feedback, and participant experiences), modules (clinician guidance and peer narratives, resources on topics related to congenital heart disease [CHD] and health system navigation), and optional connections to wearables, smartphone data, and patient portals. Participants also receive a “Poke” notification roughly every 2 months to remind them of new activities (step 4). UCSF: University of California San Francisco.

Participants can be recruited through email, clinic flyers, social media, or community outreach using QR codes. Participants can engage via smartphone or web browser and sign electronic consent. Activities are delivered bimonthly (every 2 months) and remain available for 2 months. Each activity includes surveys, educational modules, and optional wearable or portal linkages (Figure 3; Table S8 in Multimedia Appendix 1). Engagement is supported through multimodal communication (app notifications, email, texting), with up to 3 reminders per activity cycle. Bimonthly “Pokes” use variable timing and content (peer stories, study updates, CHD research opportunities) to maintain interest through intermittent reinforcement.

#### Core Intervention Components

**Figure 4.**
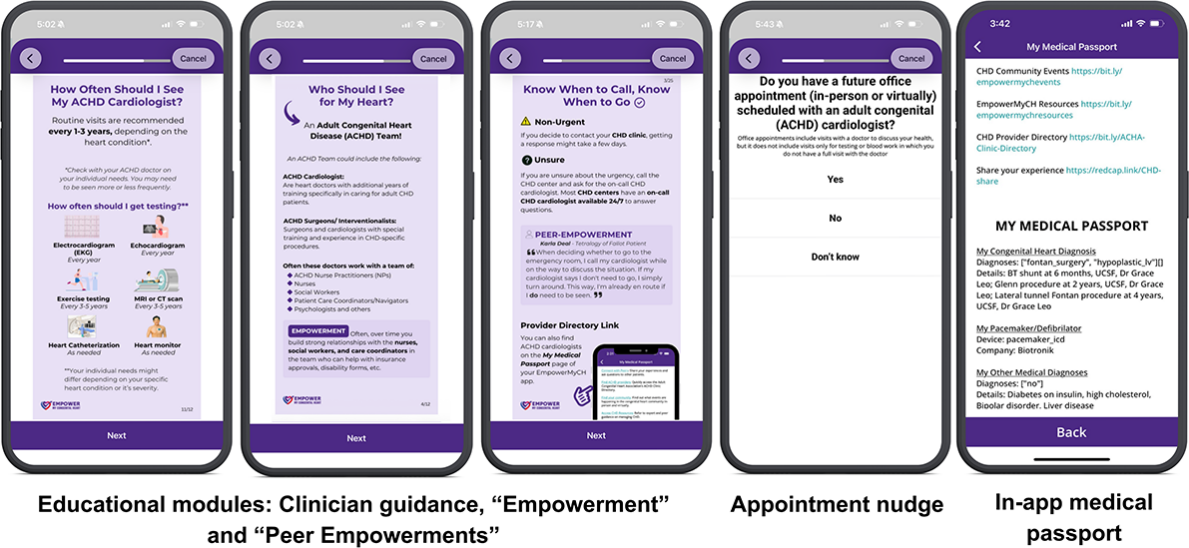
Representative core intervention components within Empower My Congenital Heart (EMCH). The screenshots show representative screens of the educational modules (with clinician guidance, “Empowerment,” and patient-derived “Peer Empowerment” stories), appointment planning nudges (embedded within the surveys), and personal digital medical passport (a centralized hub for all medical and provider information while also linking participants to curated community events, all EMCH modules, the adult congenital heart disease [ACHD] provider directory, and a secure feedback and contact form). The peer connections component of EMCH is integrated within the modules and community event listings.

**Figure 5.**
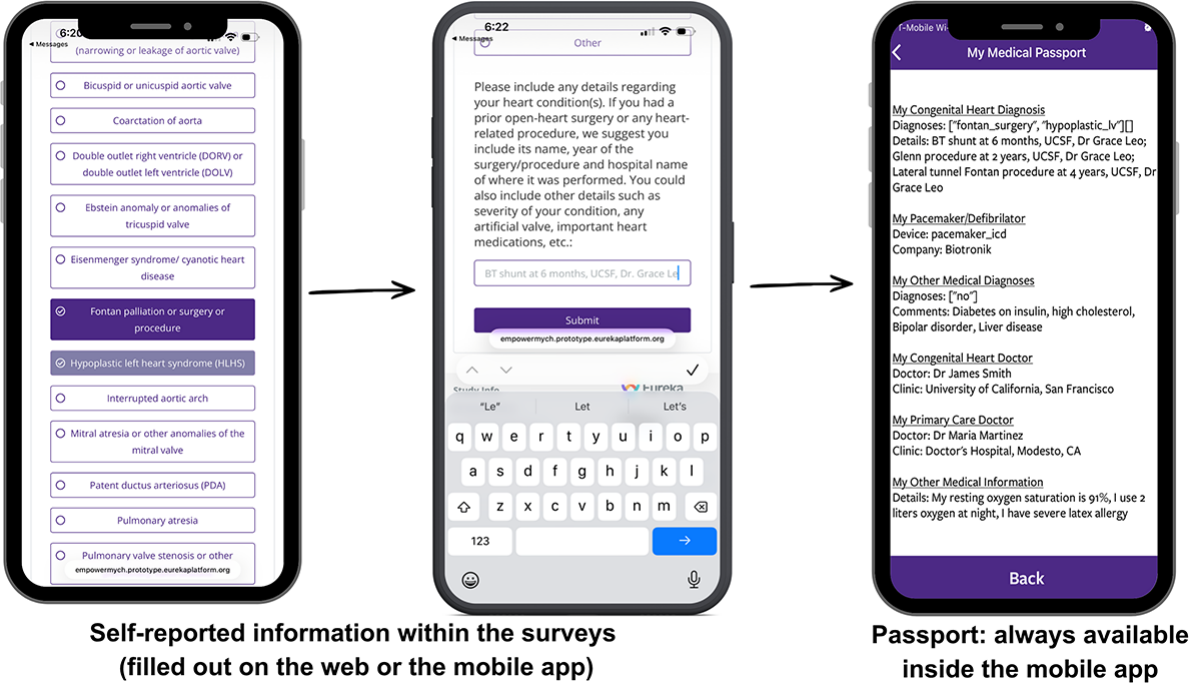
Digital medical passport built using self-reported data. This figure illustrates the type of health information easily accessible to participants at any time through the Empower My Congenital Heart (EMCH) mobile app. By completing a series of surveys with self-reported data, participants build their own digital medical passport, a personalized hub for their vital health and provider information.

The core intervention component includes educational modules (addressing *capability, opportunity, and motivation* barriers) that combine concise educational content with clinician guidance, “Empowerment” tips, and patient “Peer Empowerment” messages (Figure 4 and Multimedia Appendix 2; Table S9 in Multimedia Appendix 1) to build knowledge, confidence, and self-advocacy skills. Year 1 modules cover CHD education and health system navigation, while subsequent modules address psychosocial concerns, lifestyle, and other topics (Table S10 in Multimedia Appendix 1). Additional components include appointment planning nudges (addressing *capability* barriers) embedded within annual surveys to prompt scheduling ACHD specialist visits; personal digital medical passport (addressing *physical opportunity* barrier), auto-generated from baseline self-reported data (diagnosis, providers, etc) serving as a centralized information hub always accessible via smartphone (Figure 5; Table S11 in Multimedia Appendix 1); and peer connections (addressing *social opportunity and motivation* barriers) through vetted Peer Empowerment and community event listings, with all content reviewed for accuracy and safety before inclusion.

#### Iterative Refinements During Intervention Development

Throughout development, intervention features were refined based on pilot testing, CAB feedback, and adherence to digital health equity principles (Table 5). These refinements demonstrate the responsive, user-centered development process that shaped the final EMCH design.

**Table 5.** Iterative refinements during intervention development.

Initial design	Refinement	Rationale	Data source
Monthly delivery of the module	Modules delivered every 2 months	Reduce participant burden while maintaining engagement	CAB^a^ feedback
Colorful icons or imagery	Flat, minimalist icons	Enhance readability, reduce bandwidth needs, improve accessibility	Digital health equity principles + CAB feedback
Frequent reminders (9 total: 3 email, 3 push, 3 texts per module)	Reduced to 3 total reminders per EMCH^b^ activity	Minimize intrusiveness, respect participant time	User testing + CAB feedback
Reminders sent until all activities completed (survey and modules)	Reminders sent only if module incomplete; most surveys are not reprompted	Minimize intrusiveness for data collection while providing opportunities to engage with modules	CAB feedback
Digital passport surveys delivered at 2 months post enrollment	Digital passport surveys delivered at the time of enrollment	Maximize early engagement with digital passport from first access	User testing + CAB feedback
Clinician guidance and peer narratives labeled as “Tips”	“Clinician guidance,” “Empowerment” quotes, and “Peer Empowerment” quotes included as distinct color-coded categories	Emphasize empowerment, positivity, and a component of “expert and peer guidance” as primary intervention goals	CAB feedback
Module content primarily informational	Added interactive elements (trivia questions, action items) within the modules	Enhance engagement and encourage clear, actionable takeaways	CAB feedback
Digital passport to include comprehensive data (CHD^c^ history, other medical history, all providers, medications, insurance)	Digital passport limited to pertinent medical and provider information	Simplify content to enhance usability and relevance; reduce data entry burden	Eureka platform team + CAB feedback

a CAB: community advisory board.

b EMCH: Empower My Congenital Heart.

c CHD: congenital heart disease.

### Data Security and Privacy

The Eureka Platform uses 128-bit secure socket layer encryption for data transmission between the app and servers. Data are stored on Health Insurance Portability and Accountability Act–compliant servers managed by UCSF with restricted access, multifactor authentication, and role-based permissions. Survey responses and engagement metrics are stored as separate comma-separated values files using unique identifiers without personal identifiable information. Only the authorized research team can link the deidentified data to individual participants. Participants can withdraw at any time by contacting the research team. Upon withdrawal, they may request the deletion of their data or allow deidentified data to remain for research purposes. Participants control whether their stories can be shared as Peer Empowerment content (explicit opt-in required). Wearable device data (Fitbit) and patient portal connections require separate participant authorization and are governed by those platforms’ privacy policies in addition to EMCH protections. Study data will be retained per UCSF IRB and NIH data sharing policies, after which the data will be securely destroyed or permanently deidentified.

## Discussion

### Principal Results

We describe a theory-based, community co-designed approach to developing EMCH, a digital intervention promoting confidence and skills to enhance engagement in lifelong ACHD care. To our knowledge, this is the first cross-platform (Android and iOS) digital tool specifically designed to address ACHD-specific barriers to lifelong specialized engagement. Our approach integrated evidence-based behavioral frameworks (COM-B or BCW) with extensive qualitative research (n=54) and community-based participatory design to map barriers to engagement and develop targeted intervention components. Our approach ensured that patient and clinician perspectives shaped all design decisions. EMCH enables convenient, passive content access using user-friendly design and behavioral economics (nudge) principles while fostering peer connections and community contributions.

### Clinical Relevance and Expected Impact

Existing CHD digital interventions focus narrowly on symptom monitoring, exercise support, or transition readiness in young adults and lack theoretical grounding or rigorous evaluation [23,24]. EMCH addresses the full adult lifespan (18+ years) and targets the complete spectrum of barriers to ongoing specialist care for all patients, including those who never transitioned from pediatric care or were lost to follow-up. The intervention’s systematic foundation in behavioral theory (COM-B or BCW) distinguishes it from education-only approaches by addressing psychological capability, social opportunity, and motivation simultaneously. If effective, EMCH could reduce preventable ACHD complications, emergent hospitalizations, and mortality associated with care gaps by promoting sustained engagement with specialist care.

### Scalability and Sustainability

We aim to recruit patients with ACHD at high risk for care gaps (young adults, men, rural residence, lower socioeconomic status, those living far away from CHD centers, etc) and those not previously engaged in research. Mobile phones are nearly ubiquitous—96% of Americans (and 94% of patients with ACHD) in their twenties and thirties own smartphones, with particularly high reliance among young adults, non-Whites, and lower-income Americans [22,49,50]. EMCH cross-platform, low-bandwidth design, and digital health equity framework enable reach across diverse populations. EMCH was designed to augment, not replace, patient-provider interactions by building engagement skills outside clinic visits. Unlike clinic-based navigator programs requiring ongoing personnel costs, EMCH automated content delivery and peer-generated stories provide sustainable engagement with minimal marginal cost per additional user. We selected the Eureka Digital Research Platform for its sustainability (NIH-supported, UCSF-owned), integration of recruitment through intervention delivery, and cost-efficiency in supporting hundreds of concurrent studies.

### How EMCH Differs From Existing Approaches

We selected BCW over alternative frameworks (eg, Intervention mapping or the Consolidated Framework for Implementation Research) [51,52] because it provides flexible, evidence-based guidance for digital behavior change interventions without being overly prescriptive [25,53]. The BCW process enabled transparent documentation of our intervention’s theory of action by systematically mapping behavioral barriers to specific components using shared taxonomic language [32]. Integrating a community-engaged approach with behavioral theory proved essential for moving beyond identifying barriers [30]. While we knew many patients with ACHD lack the awareness of specialist care needs [5], interviews and CAB feedback showed that evidence-based information alone was insufficient. Participants needed peer stories and real-life scenarios to internalize the relevance. This insight led to “Empowerment” and “Peer Empowerment” quotes becoming core components, using modeling to build confidence and demonstrate care’s importance when patients feel well.

The combined qualitative, theory- and community-engaged approach can be resource-intensive but adaptable. Teams with limited resources can supplement qualitative work with rapid stakeholder consultation while maintaining the framework’s core strengths: preventing the reinvention of existing content, simultaneously addressing engagement and implementation barriers, and ensuring intervention components meet users’ actual needs.

### Limitations

EMCH addresses patient-level barriers but cannot overcome system-level challenges (ACHD specialist shortages, insurance gaps, geographic distance from centers). The intervention is most appropriate for stable patients capable of self-management with remote guidance; patients with complex acute needs or severe cognitive impairment require in-person clinical support. While 94% of patients with ACHD own smartphones [22] and EMCH is available on web and native iOS or Android platforms with low-bandwidth design, the most vulnerable patients may face barriers beyond digital intervention scope. However, by reducing routine educational burden for digitally engaged patients, EMCH may enable clinical teams to prioritize in-person resources for those with greater needs. The digital passport currently captures baseline data only; update functionality is under development, and we are exploring electronic health record integration to auto-populate verified clinical data. Although we incorporated diverse perspectives during development, we may not have captured all viewpoints. The EMCH infrastructure supports continuous adaptation, enabling rapid implementation of new components (eg, mental health modules, culturally adapted content). Currently, individuals with intellectual disabilities are excluded due to unique accommodation needs requiring caregiver-focused approaches.

### Next Steps

EMCH was launched in September 2024 and has enrolled more than 500 participants. Ongoing process evaluation is assessing acceptability, usability, and preliminary engagement patterns across diverse ACHD populations. We are developing a Spanish-language adaptation to reach the 15% to 20% of patients with ACHD who are primarily Spanish-speaking.

Following process evaluation, we will conduct a randomized controlled trial evaluating EMCH’s effectiveness in completing ACHD specialist visits and improving knowledge, self-efficacy, and social connectedness. If proven effective, EMCH will be scaled to reach patients with ACHD nationally through partnerships with community advocacy organizations and integration into diverse clinical settings.

### Conclusions

The theory-based, community co-designed EMCH digital tool equips patients with ACHD with evidence-based resources, peer support, and practical skills to sustain engagement with lifelong specialized care. Ongoing process evaluation will identify the acceptability, usability, and engagement patterns to inform a planned randomized controlled trial. If proven effective in reducing care gaps, EMCH has the potential to reduce preventable ACHD complications, emergent hospitalizations, and mortality—outcomes currently affecting the majority of patients with ACHD. The behavioral frameworks and intervention strategies address universal barriers to care engagement and can be adapted for other chronic conditions requiring lifelong specialized care. The EMCH infrastructure also supports the rapid recruitment of diverse ACHD populations for future research and health policy initiatives.

## Supplementary material

10.2196/75867 Multimedia Appendix 1 Supporting materials and tables.

10.2196/75867 Multimedia Appendix 2 Screenshots of the intervention components within Empower My Congenital Heart (EMCH).

## Abbreviations

ACHD: adult congenital heart disease
BCW: Behavior Change Wheel
CAB: community advisory board
CHD: congenital heart disease
COM-B: Capability, Opportunity, Motivation for Behavior Change
EMCH: Empower My Congenital Heart
IRB: institutional review board
NIH: National Institutes of Health
UCSF: University of California, San Francisco
