# Supplementary material for: Digital Health Intervention to Promote Lifelong Specialized Care in Adults With Congenital Heart Disease: Theory-Driven Community Co-Designed Study
Source: J Med Internet Res. 2026 Jun 23;28:e75867. doi: 10.2196/75867 (PMC13289845; doi:10.2196/75867)
Supplement: Multimedia Appendix 1 [file jmir-v28-e75867-s001.docx]

Table of Contents

[Table S1. Semi-structured Interview Guide- Patients 2](#_Toc232149593)

[Table S2. Semi-structured Interview Guide- Clinicians 4](#_Toc232149594)

[Table S3. EMCH Design Guide 5](#_Toc232149595)

[Table S4. Sample Codebook Entries Mapped to COM-B Framework 5](#_Toc232149596)

[Table S5. Sample Rapid Qualitative Analysis Matrix Structure 6](#_Toc232149597)

[Table S6. Emerging Hopes for the Digital Tool 7](#_Toc232149598)

[Table S7. Eureka Digital Research Platform Technical Specifications 8](#_Toc232149599)

[Figure S1: Digital Tool Branding Prototypes 9](#_Toc232149600)

[Table S8. Participant Flow within EMCH 10](#_Toc232149601)

[Table S9. Sample Clinician Guidance, Empowerment and Peer Empowerment Messages in EMCH 11](#_Toc232149602)

[Table S10. Sample EMCH Educational Module Topics 13](#_Toc232149603)

[Table S11. Digital Medical Passport Data Fields 14](#_Toc232149604)

# **Table S1. Semi-structured Interview Guide- Patients**

| **Questions** | **Prompts** |
| --- | --- |
| *Childhood Memories*  Growing up, what was it like to have a heart condition? | - What did you know or understand about your heart condition? How did you gain that knowledge? - What was your understanding about the need for follow up for your heart condition? - What was your understanding about any complications or problems related to your heart in the long-term? |
| *Adult Transition*  Now that you are an adult, what is it to like to have a heart condition? | - How did your knowledge of your heart condition change from when you were a child to when you were an adult? Why do you think this change occurred? - What did you understand or know about how your heart condition will be managed after adulthood? - Looking back at your own care – what would you have liked to know? What would have helped you? What would you have wanted? - What was the transition like between your child and adult heart doctor? - What were your thoughts or feelings around moving from a pediatric to adult doctor? - Have you used any resources to aid your transition to adult care? Why/why not? |
| *Navigating Health System*  What was your experience with the healthcare system like once you were an adult? | - Were there any times when it was [easy OR difficult] to make an appointment? What was that like? - Why do you think it was [easy OR challenging]? - What do you wish you knew beforehand that could have helped you? - How was your experience making an appointment with the adult health care system? - How did you decide who was the right heart doctor for you to make an appointment with? |
| *Gaps in Care*  How do you think your follow-up care for your heart disease has been throughout your adulthood? | - Tell me about your experience when you were not following up with any heart doctor regularly. What was it like? - How long was that time period? - Why do you think you experienced these gaps in care with your heart doctor? - What could have helped you stay in care? - [For no gaps in care] Why do think your follow-up care was consistent? |
| *Benefits/Consequences/Motivations of Being “In Care”*  Seeing a specialist regularly can be challenging on our schedules—what are your thoughts on the need for ongoing care? How do you manage it with your schedule? | - How often do you think someone with your condition should see their heart doctor? Who should be involved in that decision? - How do you manage visiting an ACHD specialist with your other commitments? - What do you think are the biggest challenges? How do you overcome them? - What helped or could help you feel more confident in your abilities to find specialists and schedule meetings with them? - How do you remember to schedule and make an appointment? How do you feel this system is working for you? - What could an ACHD specialist, or another member of your care team, do to make it easier to keep track of appointments? |
| *Intervention Components*  We are going to change topics slightly to talk about potential goals for your healthcare. What are your thoughts about setting some personal goals regarding your health? | - How often do you set goals in your life? What kind of goals do you set? And how do you set them? - How do you work toward achieving those goals? - How do you stay on track to meet your goals? What kinds of things help you measure your progress? - How often do you involve other people in creating or maintaining your goals? Why do you think that is? What [has OR has not] been helpful about having another person involved? What kind of support would you want from another person? - When you think of times where you didn’t reach a goal you wanted, what got in your way? - When have you felt especially motivated to achieve a goal? Where did the motivation come from and what helped maintain it? - [If participant doesn’t set goals] What do you do to achieve the outcomes you want? How has this process worked for you in the past? What do you think could make your process even more useful? |
| *App-based Solutions*  Now, I’d like to focus on some proposed technology-based solutions to possible problems patients face when navigating the healthcare system as an adult.  What are your thoughts about receiving messages through an app on a phone that can support the process of establishing care with your heart doctor? | - What are the kinds of health-related things for which you use an app on the phone? (e.g. exercise tracking, heart rate, etc.) - Can you list or show some of the common apps you use and what you use them for? - How do you see a mobile Health app being able to help you with maintaining life-long care for your heart? - What challenges could you anticipate in having an app like this? What suggestions do you have in order to make this tool easier for you? |

We’re at the end of the interview, but before we conclude I would like to hear about anything you feel we missed.

- Is there anything you’d like to tell me, that maybe I didn't ask you about, but you think it is important for me to know?
- Do you have anything that you would like to ask me/us?

Thank you for your candid responses to our questions.

# **Table S2. Semi-structured Interview Guide- Clinicians**

| **Questions** | **Prompts** |
| --- | --- |
| *Introduction to Role/Experiences*  I would like to hear about your experiences caring for patients who have *recently transferred* to adult CHD care.  *are being transferred - *pediatric providers* | - Can you tell me about your current role and the responsibilities that come with it? - How has your experience been providing [adult/pediatric] services? |
| *The CHD Team*  I would like to hear about your experiences with other clinicians in managing CHD patient care. | - Who are the people you consider to be on your team? Are they adult or pediatric based providers? - How is the communication between the different services? - What similarities or differences do you notice between the pediatric and adult services?   - How do these [similarities/differences] influence 1) how you do your job 2) how you view a patient's transition of care? |
| *Independent Decision Making*  What do you think prepares a patient to make independent decisions around their health care? | - How do you assess whether a patient has those things or that knowledge? - What interaction, if any, do you see between your role and preparing a patient to make independent decisions? - What aspects of your role may make it hard to help build this independence? |
| *Transition Facilitators*  What are services or things in the system that  help youth transition from the peds to adult  service? | - What would you consider essential, that if it were removed it would make transitioning or transferring impossible? - What, if added, would ease transitioning or transferring appropriately? |
| *Transition Barriers*  What services or policies are needed to improve the health care transition process? | - What consumes your time? - What affects families? - What are major barriers to transitioning and transfer? |
| *Points for Intervention*  What do you think could be improved with the transfer process from pediatric to adult  focused care or in the process of making patients more independent in making their health-related decisions? | - Are there issues to be solved with the subspecialty services? - Are there issues to be resolved with the outpatient primary care services? - Are there issues to be resolved with the outpatient subspecialty services? - How about community-based services, or other ancillary services? (PT/OT, etc.) |
| *Intervention Components*  What can help patients feel more engaged with their healthcare? | - Are there any tools or resources that could be helpful? - What and when would be the most appropriate way to deliver those resources? - How are your thoughts about patients using a mobile app? - What features of the app would be most useful? - What resources are available and can be used to help patients via app? |

# **Table S3. EMCH Design Guide**

| **Section Statements** | **Supporting Narratives** |
| --- | --- |
| Messaging statement | To empower CHD patients to proactively participate in their health journey and thrive: Every hero embarks on a journey marked by challenges and uncertainties. For patients with congenital heart disease the journey through the healthcare system can often feel daunting and overwhelming. Just as every hero experiences moments of confusion and struggle, CHD patients often find themselves navigating complex medical landscapes and decisions.  By framing the patient's experience within this heroic narrative, we ensure that our guidance is both empowering and supportive. |
| Design statement | We value **readability** above all else. These design guidelines serve as a foundation for consistency and structure; if a rule compromises the clarity of the content, make exceptions to improve the design. If an element doesn't look correct or detracts from the user experience, adjust the design rather than adhere strictly to the rules. |
| Written language statement | We build our tools around the written word; we have an incredible opportunity to convey our message with precision and impact. Clarity, simplicity, and brevity are essential to ensuring that our message resonates with and reaches the appropriate audience. Every word choice is critical—each must be deliberate, contributing to a message that is both powerful and **accessible**. We prioritize **readability** in both our language and design—no amount of design can compensate for poorly crafted language. To support this, we can use tools like Simple Measure of Gobbledygook ([SMOG](https://www.ncbi.nlm.nih.gov/pmc/articles/PMC5764592/)), which estimates the readability of text by determining the grade level needed to understand it based on the frequency of polysyllabic words. By focusing on the craft of language, we can balance style and substance, leading to consistently clear and effective communication that can meet these readability standards. |

# **Table S4. Sample Codebook Entries Mapped to COM-B Framework**

| **Theme/Code** | **Definition** | **COM-B Domain** | **Representative Quotes** |
| --- | --- | --- | --- |
| Knowledge gap about CHD condition | Participant lacks understanding of CHD diagnosis, severity, implications, or need for specialist care | Capability (Psychological) | “I didn’t really know anything about it [heart condition].’ |
| Forgetting to schedule appointments | Unintentional failure to schedule or attend recommended ACHD specialist visits | Capability (Psychological) | "I think it's less of an explicit moment-to-moment identification of I'm not going to get care, and more of just like explicit forgetting to make that appointment, because there's just an 'ugh' feel around it” |
| Difficulty finding ACHD specialist | Unable to locate, identify, or access adult congenital heart disease cardiologist | Opportunity (Physical) | "It wasn’t until I graduated college, got my own job that… I really understood how difficult it was then to get a cardiologist” |
| Feeling isolated due to rare condition | Sense of being alone in health journey; lack of connection with others who have CHD | Opportunity (Social) | "Since I have a heart disease, I always try to keep my distance. I keep my distance from my family, my friends, and sometimes that community" |
| Lack of self-advocacy skills | Difficulty communicating needs, asking questions, or advocating for oneself with healthcare providers | Opportunity (Social) | "There's a lot of people that don't know how to get the help. They don't speak up, or they just don't know that the help is there." |
| Avoidance of thinking about condition | Active or passive avoidance of CHD-related thoughts to reduce anxiety or distress | Motivation (Automatic) | "Some periods have largely been because I just didn't want to know anymore. It was more stressful to deal with, actually, dealing with the issue" |
| Codes were developed inductively from interview transcripts and subsequently mapped to COM-B domains. This mapping enabled systematic identification of barriers amenable to digital intervention and informed selection of appropriate BCW intervention functions. | | | |

# **Table S5. Sample Rapid Qualitative Analysis Matrix Structure**

| **Participant ID** | **Capability Barriers** | **Opportunity Barriers** | **Motivation Barriers** | **Digital Tool Feature Preferences** |
| --- | --- | --- | --- | --- |
| UCSF_001 | Knowledge: "It's not something I think about daily" | Physical: Limited access to specialist information | Reflective: Low urgency - "it's not pressing" | "Easy access to credible resources" |
| UCSF_003 | Knowledge: Condition understanding gaps | Social: Isolation - "I keep my distance from family and friends" | Automatic: Fear/avoidance - "Sometimes I want to give up" | "Community support and peer connections" |
| UCSF_006 | Memory: Forgetfulness not intentional | Physical: None identified | Reflective: Condition acceptance - "it's part of who I am" | Reminders and appointment nudges" |
| This table illustrates how the Rapid Qualitative Analysis matrix organized coded data across participants to identify patterns in barriers (mapped to COM-B domains) and preferences for digital tool features. Full matrix included all 54 participants. | | | | |

# **Table S6. Emerging Hopes for the Digital Tool**

| **Themes** | **Supporting Narratives** |
| --- | --- |
| Easy access to credible resources | “I think any kind of connection or links that actually get you to advocacy, or insurance companies, or softwares. Or at least kind of give information on how to navigate those things maybe in a step-by-step way with resources for that.” – Patient, Woman in their 30’s  “just being instructed on how to search for the right healthcare, that went a long way. Versus having to search it up on Google.” - Patient, Man in their 20’s  “I’ve got questions, millions of questions. And it doesn’t seem like there’s anyone that can answer them. It doesn’t feel like there’s any... At least, no one’s told me any resources that I can reach out to” - Patient, Woman in their 30’s  “it’s hard for me to understand: am I doing well or not doing well? Right? I have my annual visits with my cardiologist. Outside of that, I really don’t have a lot of other insights.” – Patient, Man in their 50’s |
| Uplifting of patient voices | “community building within the congenital heart disease community, like being able to talk to other patients. I can help younger patients who maybe are in college or went through what I had to go through in college, help them find care or also look for older patients and just find mentors.” - Patient, Woman in their 20’s  “having a community board or something, where it's like, "I was diagnosed with ASD at this point, and my favorite resource is" – that's where people, I think, connect. Everything is about human connection. “ - Patient, Woman in their 30’s  “And I think that’s what we’ve seen in our advocacy work is I think when you combine patients and doctors, you get the full experience….So, I think if your app. offered that, that would be intriguing of, “Oh, wow. Okay. I’m getting it from every point of view.” - Patient, Woman in their 20’s |
| Customization to patient needs | “I think an app would be very helpful in sharing awareness [of]... the range of CHD symptoms because we group CHD a lot, as we should, but then there’s also that CHD that exists on a spectrum.” - Patient, Man in their 20’s  “I would love to have a drawing of my condition. So, that way, if I went to another doctor, I’m like, “Here. This is what my heart looks like.” It would be good to have like a full list of here’s all your conditions. Here’s all the meds you’re on. So, it would be almost like your medical health record, essentially, like right there in your phone would be really helpful to have. But I think something specifically for your heart condition would be really, really nice.” - Patient, Woman in their 30’s  “I think what's gonna make things more successful is very individualized and localized types of tools.” - Clinician |
| Centering positivity and joy | “It’s colorful and it’s positive.” - Patient, Woman in their 50’s  “I'm envisioning something that's bright and friendly” - Patient, Woman in their 20’s  “maybe having something dedicated to CHD [meet ups]. You know, ‘anybody want to grab a drink or a coffee or whatever and talk?’... like if you sign up for the app, you can see other CHD patients around you, and maybe meet up,” - Patient, Woman in their 30’s |

# **Table S7. Eureka Digital Research Platform Technical Specifications**

| **Feature** | **Description** |
| --- | --- |
| Platform Name | Eureka Digital Research Platform (University of California, San Francisco) |
| Funding Support | National Institutes of Health/NIBIB (3U2CEB021881-05S1) |
| Infrastructure | Cloud-based (Amazon Web Services), HIPAA-compliant servers with restricted access |
| Data Security | SSL/TLS encryption for data transmission; AES-256 encryption for data at rest; multi-factor authentication for research team access |
| Access Methods | Web browser (desktop/laptop); Native iOS app (Apple App Store); Native Android app (Google Play Store) |
| Registration Process | 1. Create UCSF Eureka Research account (name, email, date of birth)  2. Verify email address and/or phone number  3. Review and sign electronic informed consent (eConsent)  4. Complete eligibility screening questions  5. Access EMCH study activities |
| Data Collection Capabilities | Surveys and questionnaires; Integration with wearable devices (Fitbit, Apple Health, Google Fit); Patient portal connections (with authorization); Push notifications, email, and SMS messaging |
| Intervention Delivery | Timed release of educational content; Multi-modal notifications; File attachments (PDFs, images); Video embedding capability |
| Data Storage and Retention | De-identified data stored separately from personally identifiable information (PII); Role-based access controls; Data retention per UCSF IRB and NIH requirements |
| Participant Data Control | Participants can withdraw at any time via app or by contacting research team; Upon withdrawal, participants can request data deletion or allow de-identified data to remain for research |
| The Eureka platform supports hundreds of concurrent digital health studies, enabling cost-efficient infrastructure sharing while maintaining study-specific data security and participant privacy protections. | |

# **Figure S1: Digital Tool Branding Prototypes**

Four logo and name combinations developed through iterative community advisory board discussions. Option B (EMPOWER my CONGENITAL HEART) with logo #2 was selected through CAB voting process for its empowering tone and upward arrow symbolizing patient activation.

# **Table S8. Participant Flow within EMCH**

The intervention design enables recruitment through email, clinic flyers, social media, or community outreach using study-specific QR codes. After registration and electronic consent, participants engage via smartphone or web browser. Activities are delivered every 2 months, a schedule selected by the CAB to balance engagement and participant burden. Each activity cycle remains available for 2 months and includes surveys, educational modules, and optional linkages to wearables, smartphone data, and patient portals (**Main Text,** **Figure 4**). Surveys gather self-reported data and feedback and allow participants to share stories or questions (**S8**). Modules provide concise guidance from ACHD experts and patients to enhance confidence and knowledge in navigating CHD care. Optional linkages enable participants to share their health data, supporting research and understanding of ACHD patient experiences and behaviors. For example, during the first 2-month cycle (enrollment to month 2), participants complete baseline demographic surveys, after which Module 1 (A Guide to Choosing Your Care Team) and optional linkages become accessible. During months 2-4, quality-of-life surveys become available, followed by Module 2 (Navigating Cardiologist Appointments) and optional linkages, if not already completed.

| **EMCH Surveys** | |
| --- | --- |
| **Domain** | **Individual Variables** |
| **Participants’ Data** | |
| Demographics | Age, sex, race/ethnicity, educational status, household income (as a measure of SES), occupation, marital and parental status |
| Diagnosis | Self-reported CHD condition and surgeries, comorbidities |
| Service Use | Outpatient primary care and cardiologist visits, emergency care use or hospitalizations |
| Patient-Reported Outcomes (PROs) | Euro QoL, Visual Analogue Scale, Health behaviors survey, Illness Identity Survey, self-reported NYHA, Generalized anxiety disorder, PHQ-8, Social history |
| COM-B Patient Engagement Domains | Patient Activation Measure (13-item, score 0-100; Cronbach’s alpha 0.91)  Gothenburg Empowerment Scale (15-item, score 15-75) |
| Participants’ Feedback | |
| Intervention Characteristics | Client satisfaction survey-8 (8 to 32, ~5 minutes, > 90% usual completion rate)  Feedback on each peer and expert advice  Regular app data download to assess use and engagement with various intervention components |
| Participants’ Personal Stories or questions | |
| Patient Engagement Survey | Opportunity for participants to ask questions or share their insights about managing CHD or connecting with peers. |

# **Table S9. Sample Clinician Guidance, Empowerment and Peer Empowerment Messages in EMCH**

| ***Sample Clinician Guidance Quotes*** |
| --- |
| “CHD doctors often face problems when medical tests, even at well-known adult centers, are unclear, incomplete, or incorrectly read. This might require them to order the same test again, causing delays with insurance, extra radiation exposure, or delaying treatment.”  “We recommend adding our 24/7 contact number to your emergency medical information. You can store it in your smartphone's emergency medical info, which can be accessed without a passcode, or link it to a medical alert bracelet. Most patients find their smartphones the easiest option.”  “Your dental care may need to be postponed following a procedure; many patients have to wait 6 months after a procedure.”  “Movement is medicine. Regular exercise is as important as any prescription in maintaining a healthy heart. Grab a friend, find an activity you enjoy, and get out there!”  “For those who typically wear breast/chest supporting garments and are scheduled to undergo a mid-sternal incision, consider bringing comfortable, supportive garments with front closures.”  “Having a pacemaker does not limit the ability to do a stress test or affect the accuracy of the results. Stress tests are ordered for various reasons—from assessing one’s exercise capacity, blood pressure and heart rate response to exercise, or signs of compromised blood supply to the heart. Ask your physician to explain why they ordered the test.” |
| ***Sample ‘Empowerment’ Tips*** |
| Often, over time you build strong relationships with the nurses, social workers, and care coordinators in the team who can help with insurance approvals, disability forms, etc.  It is important to confirm whether any variations from test to test reflect actual differences in your condition. Variations can sometimes reflect changes in the technique used to complete the study or report the test results. So, schedule a follow-up appointment with your ACHD team to discuss your test results.  If you’re prescribed antibiotics to treat a fever, you can insist on a blood culture BEFORE taking them. This ensures doctors can identify the specific bacteria if you have an infection.  MRIs could be very loud and long. Wear headphones during the test. The MRI technologists usually offer them, or you may be able to use your own during the test.  Talk to the social or case worker at your primary care or CHD clinic for help finding transportation resources in your area and assistance with applying.  When completing paperwork for college, you may be asked if you want to enroll in the college's student health insurance plan. This can be a good option for you if you aren’t able to stay on your parents’ healthcare plan or don't have an insurance plan. |
| ***Sample Patient ‘Peer Empowerment’ Quotes*** |
| “When deciding whether to go to the emergency room, I call my cardiologist while on the way to discuss the situation. If my cardiologist says I don't need to go, I simply turn around. This way, I'm already en route if I do need to be seen.”.  “If you have young children, consider reading books like My Scar is Beautiful to prepare them.”  “It may not come to mind but ask your medical team for advice on navigating physical intimacy with yourself and/or partners after surgery and maintaining connection in new ways.”  “I was concerned when I had an allergic reaction to contrast because I did not know if the test would still be meaningful without the dye. I was happy to learn that these tests are still meaningful even if your provider has to take precautions.”  “When my insurance company denied a test ordered by my cardiologist. I spoke with my cardiologist, and we discussed two possible solutions. The first option was to request a Peer-to-Peer Review. The second, suggested by my cardiologist, was to postpone the test to a later date when my insurance company would most likely approve it. I decided to postpone the test and have a virtual visit with my cardiologist instead.”  “You should not be concerned if the test is longer, shorter, or sounds different than other tests you've taken. The experience can vary considerably based on the sonographer, their equipment, and the part(s) of the heart they are observing.”  “I live a normal life, but I have to be proactive in caring for myself to ensure the best quality of life possible. I think the anxiety associated with having my condition should not be understated.”  “My mental health toolkit centers around journaling and going to church.”  “I think it's important to know it's ok and valid to feel how you feel about having a CHD. Everyone's perspective, background, and history are different, so it's only natural that how we manage life with a CHD will be different too.” |
| *All messages are integrated as relevant within the content of the modules, pokes and other materials.* |

# **Table S10. Sample EMCH Educational Module Topics**

| **Module Title** | **Primary COM-B Target** | **Key Learning Objectives** |
| --- | --- | --- |
| Choosing My ACHD Team | Capability (Knowledge); Opportunity (Environmental context) | Differentiate ACHD specialists from general cardiologists; introduce ACHD clinic directory |
| Navigating My Cardiologist Appointment | Opportunity (Social- self-advocacy) | Provide strategies for effective communication with providers |
| Navigating My CHD Tests | Capability (Knowledge) | Explain common cardiac tests and how to interpret results |
| Symptom Awareness and Action | Capability (Knowledge); Motivation (Beliefs about consequences) | Distinguish urgent vs. non-urgent symptoms; provide action plan |
| Approaching CHD Procedures | Capability (Knowledge);  Opportunity (Social- self-advocacy) | Explain common CHD procedures (catheterization, surgery); describe what to expect and questions to ask providers |
| Mental Health Toolbox | Opportunities (Environmental resources);  Motivation (Optimism) | Normalize mental health challenges in CHD; introduce coping strategies and professional mental health resources |
| Exercise and CHD | Opportunities (Environmental resources);  Motivation (Optimism) | Promote safe physical activity within individual constraints; address activity limitations and cardiovascular health maintenance |
| Lifestyle Habits and CHD | Motivation (Goals) | Identify modifiable lifestyle factors (diet, sleep, stress management) that support heart health; set achievable health goals |
| Understanding CHD Conditions* | Capability (Knowledge);  Opportunity (Environmental resources);  Motivation (Beliefs about consequences) | Explain CHD diagnosis using accessible analogies; provide guideline recommendations for follow up; explain lesion-specific long-term complications and management options |
| Navigating Life Changes With CHD | Opportunity (Social influences);  Motivation (Confidence) | Address CHD considerations during major life transitions (career, relationships, moving); build confidence in managing CHD across life stages |
| CHD and comorbidities* | Capability (Knowledge);  Motivation (Beliefs about consequences) | Explain common comorbid conditions (arrhythmias, heart failure, liver disease); describe integrated management approaches |
| *Allows self-directed personalization where participants can select and access only relevant content. | | |

# **Table S11. Digital Medical Passport Data Fields**

| **Section** | **Data Field Collected** |
| --- | --- |
| *Auto-generated Content from self-reported data and displayed in the “My Medical Passport” tab of the App* | |
| My Congenital Heart Diagnosis | Specific heart defect; free text to describe details such as surgical or procedural history |
| My Pacemaker/Defibrillator (if applicable) | Device type, manufacturer |
| My Other Medical Diagnoses | Medical comorbidities (diabetes, hypertension, arrhythmias, etc.), free text to describe details |
| My Congenital Heart Doctor | ACHD cardiologist name and clinic location |
| My Primary Care Doctor | Primary care doctor name and clinic location |
| My Other Medical information | Free text to add details about their medical condition that patients prefer to keep them easily accessible (e.g. baseline oxygen saturation levels, allergies, anticoagulation details, etc.) |
| *Content Always Available on the “My Medical Passport” tab of the App* | |
| CHD Community Events | A link to curated community events, frequently updated by our research team |
| EMCH Resources | A link to access all the EMCH educational modules |
| CHD Provider Directory | Adult Congenital Heart Association’s Clinic directory for patients to find ACHD physicians by their location |
| Share your experience | Link to a secure form to share their lived experience as an ACHD patient |
| Passport data is self-reported at baseline. Users are encouraged to verify accuracy with their ACHD care team and can request updates by contacting the research team. The passport is accessible via smartphone and can be displayed to providers during clinic visits or emergency situations. | |
