# Supplementary material for: Digital Health Intervention to Promote Lifelong Specialized Care in Adults With Congenital Heart Disease: Theory-Driven Community Co-Designed Study
Source: J Med Internet Res. 2026 Jun 23;28:e75867. doi: 10.2196/75867 (PMC13289845; doi:10.2196/75867)
Supplement: Multimedia Appendix 2 [file jmir-v28-e75867-s002.pdf]

Sample Medical Passport Screenshots

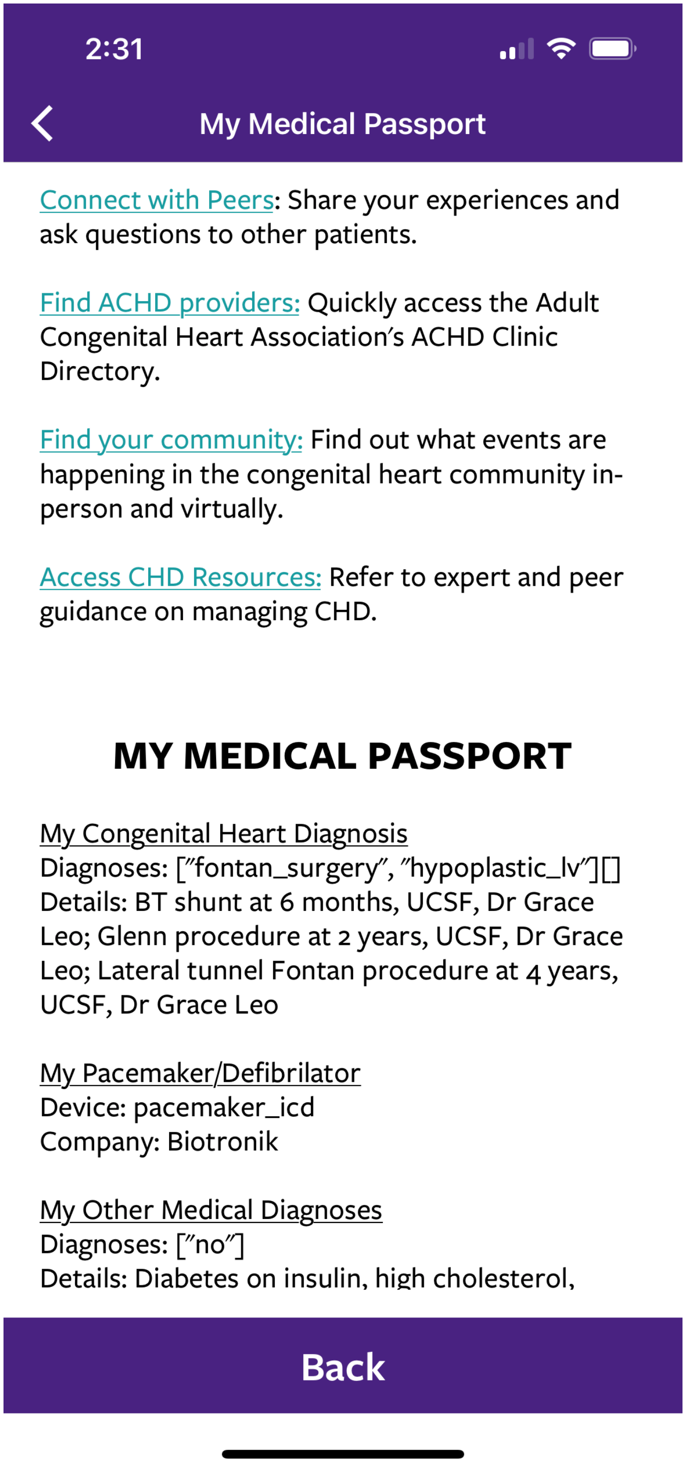

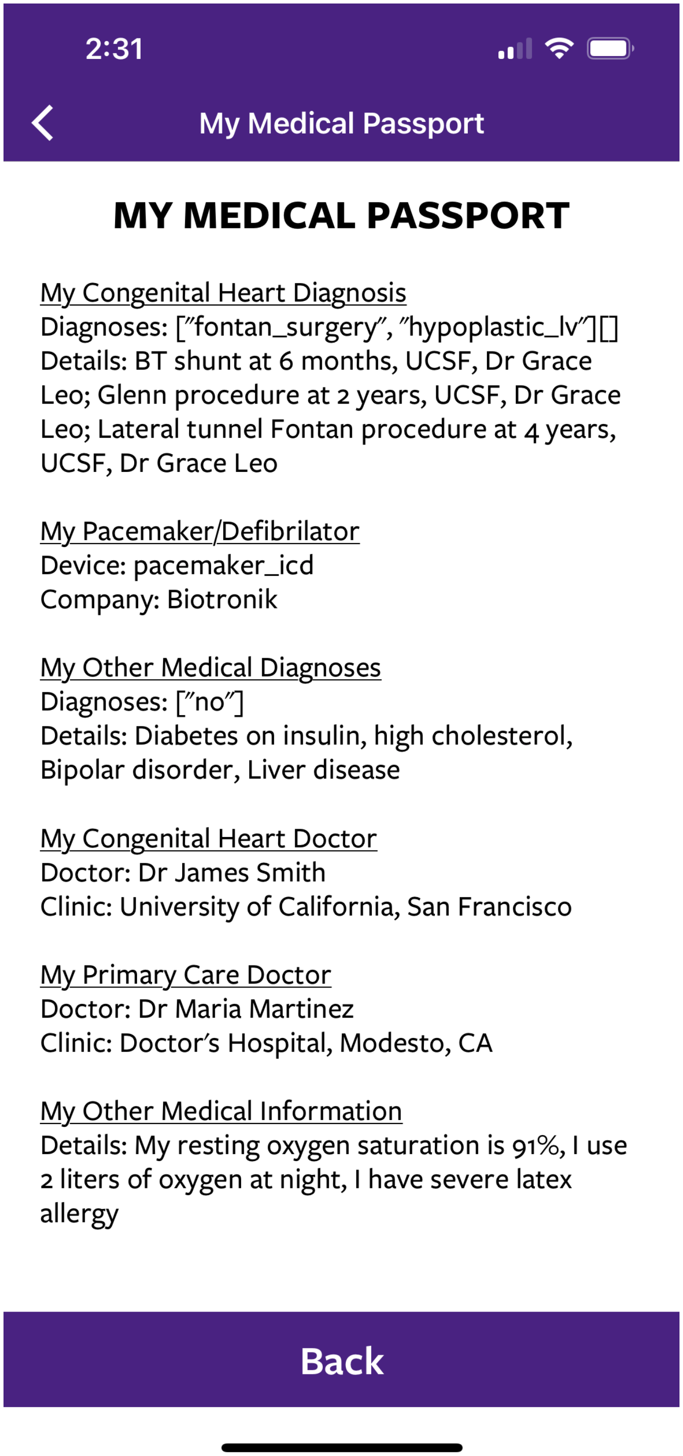

Representative Educational Module Pages

Defect vs. Disease

Congenital heart defects and congenital heart disease are closely related, but they are not the same.

Congenital Heart Defect

A congenital heart defect is a **structural problem** in the heart that is present from birth. These defects can often be **repaired** to **improve** heart function.

Congenital Heart Disease

Congenital heart disease refers to the **long-term effects** that these heart defects have on your heart health. Even after repairs, your heart will need ongoing **specialized care** and monitoring **throughout your life**.

STRUCTURAL → THE FRAME

- Think of your **heart** as the **structure or walls** of a house.
- The **chambers** are like **rooms**, each with its own purpose (the atria receive blood, and the ventricles pump it out).
- The **valves** are like your **doors** between rooms that only open one way, keeping blood moving in the right direction.
- The **septum** (the wall between the right and left sides) is like the **dividing wall** that keeps rooms separate.

When there's a "hole in the wall," like a septal defect, it's like a leak between your cold garage and warm bedroom. Air mixes, and your heating system has to work harder to keep the house comfortable.

Example of a Defect Causing a "Hole in the Wall"
Atrial Septal Defect (ASD)

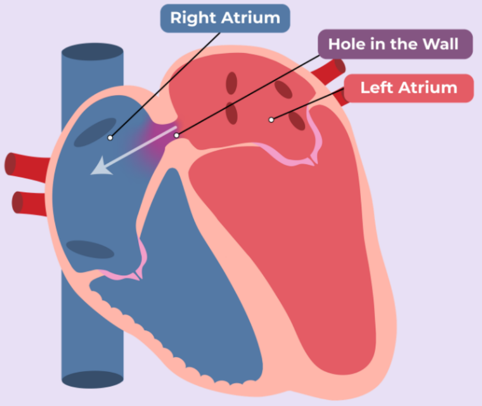

Representative Educational Module Pages

PLUMBING → THE PIPES

- The **arteries and veins** are like the **plumbing pipes** that carry water through the house.
- **Arteries** carry blood **away** from the heart, like pipes that carry water out of the house.
- **Veins** bring blood **back**, like water lines that carry water into the house.
- The **lungs** act like a water **filter**; they refresh the blood with oxygen before it returns to the heart.

If the plumbing (a blood vessel) is narrowed or blocked, it's like a clogged pipe, causing pressure to build and blood flow to slow.

Example of a Defect Causing a Narrowed Pipe
Coarctation of the Aorta

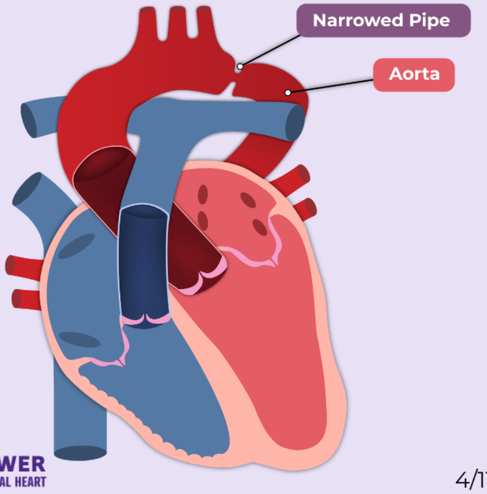

BRINGING IT ALL TOGETHER

The **Structure** of the heart gives it its shape and guides the flow of blood.

The **Plumbing** (the blood vessels) moves blood throughout the body.

The **Pump** (the heart muscle) maintains strong blood flow.

The **Electrical** signals keep the heartbeat in rhythm.

The **Valves** control the direction of blood flow.

Representative Educational Module Pages

Know When to Call, Know When to Go

⚠ Non-Urgent

If you decide to contact your **CHD clinic**, you might not get a response for a few days.

? Unsure

If you are unsure about the urgency, call the CHD center and ask for the on-call CHD cardiologist. Most **CHD centers** have an **on-call CHD cardiologist available 24/7** to answer questions.

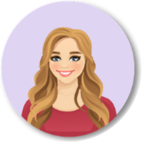

PEER EMPOWERMENT

***Karla Deal**, Patient with Tetralogy of Fallot*

“When deciding whether to go to the emergency room, I call my cardiologist while on the way to discuss the situation. If my cardiologist says I don't need to go, I simply turn around. This way, I'm already en route if I **do** need to be seen.”

Provider Directory

You can also find ACHD cardiologists on the ***My Medical Passport*** page of your EmpowerMyCH app.

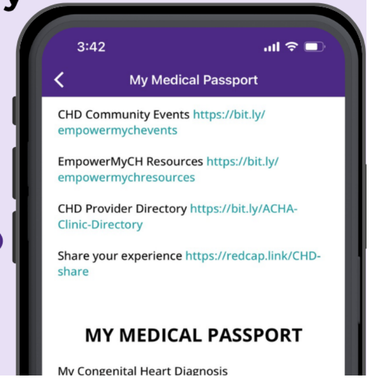

EMPOWER MY CONGENITAL HEART

Triage Guide: Shortness of Breath or Difficulty Breathing

✅ Monitor at Home (Call if Symptoms Persist or Worsen)

- Mild shortness of breath that happens only with exercise but improves with rest.
- Temporary shortness of breath due to anxiety or a mild cold, but no other concerning symptoms.

CLINICIAN EMPOWERMENT

***Mark Norris**, MD, MS*
ACHD Cardiologist

“We recommend adding our 24/7 contact number to your emergency medical information. You can store it in your smartphone's emergency medical info, which can be accessed without a passcode, or link it to a medical alert bracelet. Most patients find their smartphones the easiest option.”

EMPOWER MY CONGENITAL HEART

Representative Educational Module Pages

Triage Guide

Shortness of Breath or Difficulty Breathing

Seek Emergency Care (Call 911 or go to the ER) If You Have:

- Severe shortness of breath that happens suddenly or gets worse quickly.
- Trouble breathing at rest (even when sitting or lying still).
- Chest pain, pressure, or tightness, along with shortness of breath.
- Bluish lips, face, or fingertips (signs of low oxygen) that are different or worse from your baseline.
- Feeling dizzy, fainting, or confusion with breathing trouble.
- Coughing up pink or foamy mucus (sign of fluid in the lungs).

Contact Your Cardiologist Promptly If You Have:

- Gradual worsening of shortness of breath over days or weeks.
- Increased difficulty breathing during baseline activities (e.g., walking short distances, climbing stairs).
- Needing more pillows to sleep or waking up gasping for air.
- New or worsening swelling in the feet, legs, or stomach, along with shortness of breath.

Who Should I See for My Heart?

An Adult Congenital Heart Disease (ACHD) Team!

An ACHD Team could include the following:

ACHD Cardiologist:
Heart doctors with additional years of training specifically in caring for adult CHD patients.

ACHD Surgeons / Interventionalists:
Surgeons and cardiologists with special training and experience in CHD-specific procedures.

These doctors may work with a team of:

- ACHD Nurse Practitioners (NPs)
- Nurses
- Social Workers
- Patient Care Coordinators/Navigators
- Psychologists
- And more

EMPOWERMENT

Over time, you often build strong relationships with the **nurses, social workers, and care coordinators** on the team who can help with insurance approvals, disability forms, etc.

Representative Educational Module Pages

What Should I Look for in Imaging Reports?

2 **Structural Details:** How the heart structures appear, such as narrow or enlarged blood vessels.

3 **Focus on the Change:** You have lived with your CHD all your life. So, how your heart functions or its structure appears on one report is less important than what has changed over time. Discuss any changes with your cardiologist, noting if new or worsening symptoms accompany them.

EMPOWERMENT

It is important to confirm whether any variations from test to test reflect actual differences in your condition. Variations can sometimes reflect changes in the technique used to complete the study or report the test results. So, schedule a follow-up appointment with your ACHD team to discuss your test results.

What Should I Look for in Imaging Reports?

There are many nuances to how a report is generated. Below is a general guide.

1 **Heart Function Details:** Your Echo, MRI, or CT report will often contain a summary of your heart valves and heart function, including:

- **Stenosis:** Describes a valve narrowing that can restrict blood flow. Pressure gradient across the valve is often used to assess. *(e.g., 20 mmHg)* ***Lower numbers are usually better.***
- **Regurgitation/Insufficiency:** Describes the percentage of blood that leaks backward through a valve. *(e.g., 10%)* ***Lower numbers are usually better.***
- **Ejection Fraction (EF):** Measures how much blood your heart pumps each beat. (e.g., 50%) ***Higher numbers are usually better.***
- **Chamber Size:** Measures how big or small the atria or ventricles are. It is often assessed by measuring the volumes. *(e.g., LVEDV, RVEDV, LAV)** ***Lower numbers are usually better.***

**Sometimes, these details are described subjectively, such as “normal”, “mild”, “moderate”, or “severely abnormal”.*
